# Von Hippel-Lindau Syndrome: Multi-Organ Involvement Highlighting Its Diverse Clinical Spectrum in Two Adult Cases

**DOI:** 10.7759/cureus.9402

**Published:** 2020-07-26

**Authors:** Balveen Singh, Monika Singla, Romil Singh, Sawai Singh Rathore, Animesh Gupta

**Affiliations:** 1 Neurology, Dayanand Medical College and Hospital, Ludhiana, IND; 2 Internal Medicine, Metropolitan Hospital, Jaipur, IND; 3 Internal Medicine, Dr. Sampurnanand Medical College, Jodhpur, IND; 4 Neurology, University of New Mexico School of Medicine, Albuquerque, USA

**Keywords:** von hippel lindau, hemangioblastoma, renal cell carcinoma, pheochromocytoma

## Abstract

There is an assortment of disorders that have multisystem involvement. Von Hippel-Lindau (VHL) syndrome, a rare autosomal dominant disease, falls in that category. VHL syndrome is associated with the formation of benign and malignant tumors in the central nervous system (CNS), adrenal gland, kidney, and eyes. In this report, we present two unusual cases of VHL syndrome presenting with multisystem engagement. The first case is of a 27-year-old male exhibiting multiple manifestations, which included hemangioblastoma of the spine, pheochromocytoma, pancreatic cyst, and retinal hemangioblastoma. The second case pertains to a 25-year-old male with various presentations ranging from retinal hemangioblastoma and pancreatitis to spinal and cerebellar hemangioblastoma. These cases emphasize the value of radiologic imaging and genetic assessment early in life when the presentation of the disease is in its preliminary stage. When an individual presents with a condition characterized by unexplained multifarious organ involvement of CNS, adrenal glands, and kidneys in the span of a few years, a differential diagnosis of VHL syndrome should be considered.

## Introduction

Von Hippel-Lindau (VHL) disease is a rare tumor syndrome of autosomal dominant inheritance involving multiple organ systems [1]. The genetic alteration responsible for this syndrome is a mutation in both of the alleles of the VHL gene located on third chromosomes' short arm. VHL syndrome patients inherit a single parental mutant VHL allele alone and develop the disease when a second wild copy is disabled or lost [2]. VHL syndrome is a rare genetic disorder distinguished by the fluid-filled visceral cyst and benign tumor in a variety of organ systems with a propensity for malignant transformation. Some of the most prevalent VHL-linked tumors are central nervous system (CNS) hemangioblastomas, neuroendocrine tumors, pheochromocytomas, retinal angiomas, middle ear tumors, and renal cell carcinomas (RCC) [3].

VHL syndrome is classified into three types: types 1, 2, and 3, and type 2 is further subdivided into types 2a, 2b, and 2c. In type 1, there is a high probability of RCC and a low probability of pheochromocytoma. Type 2a and type 2b are highly vulnerable to both RCC and pheochromocytoma. Type 2c carries a high risk of pheochromocytoma, while type 3 has a risk of Chuvash polycythemia [4]. The most frequent symptom is cerebellar hemangiomas, and they are seen in 60-80% of patients presenting with VHL [5,6]. RCC is one of the leading causes of death in VHL syndrome patients [7].

VHL is diagnosed based on clinical suspicions and confirmation by molecular testing and imaging techniques. About 50-60% of VHL patients experience the symptoms during adulthood by the age of 25. However, screening and supervision for the development of these lesions should begin in the pediatric years of patients to prevent complications and outcomes [8]. That being said, VHL is associated with an extensive range of symptoms and is difficult to diagnose early [6]. The patients present with varying symptoms in different age groups, ranging from pediatric to early adulthood phases of life. All the tumors are rarely present in one patient. The two cases we discuss in this report are unique in the sense that multi-organ system tumors, including CNS hemangioblastoma, retinal angioma, pheochromocytoma, and pancreatic islet tumor, were present in both of them.

## Case presentation

Case 1

First Incidence

In 2008, a 27-year-old Southeast Asian male with no past medical history presented with a complaint of subacute onset of blurring of vision in his right eye, which later progressed to complete loss of vision in the same eye within 12 months of the onset of symptoms.

Second Incidence

**Figure 1:**
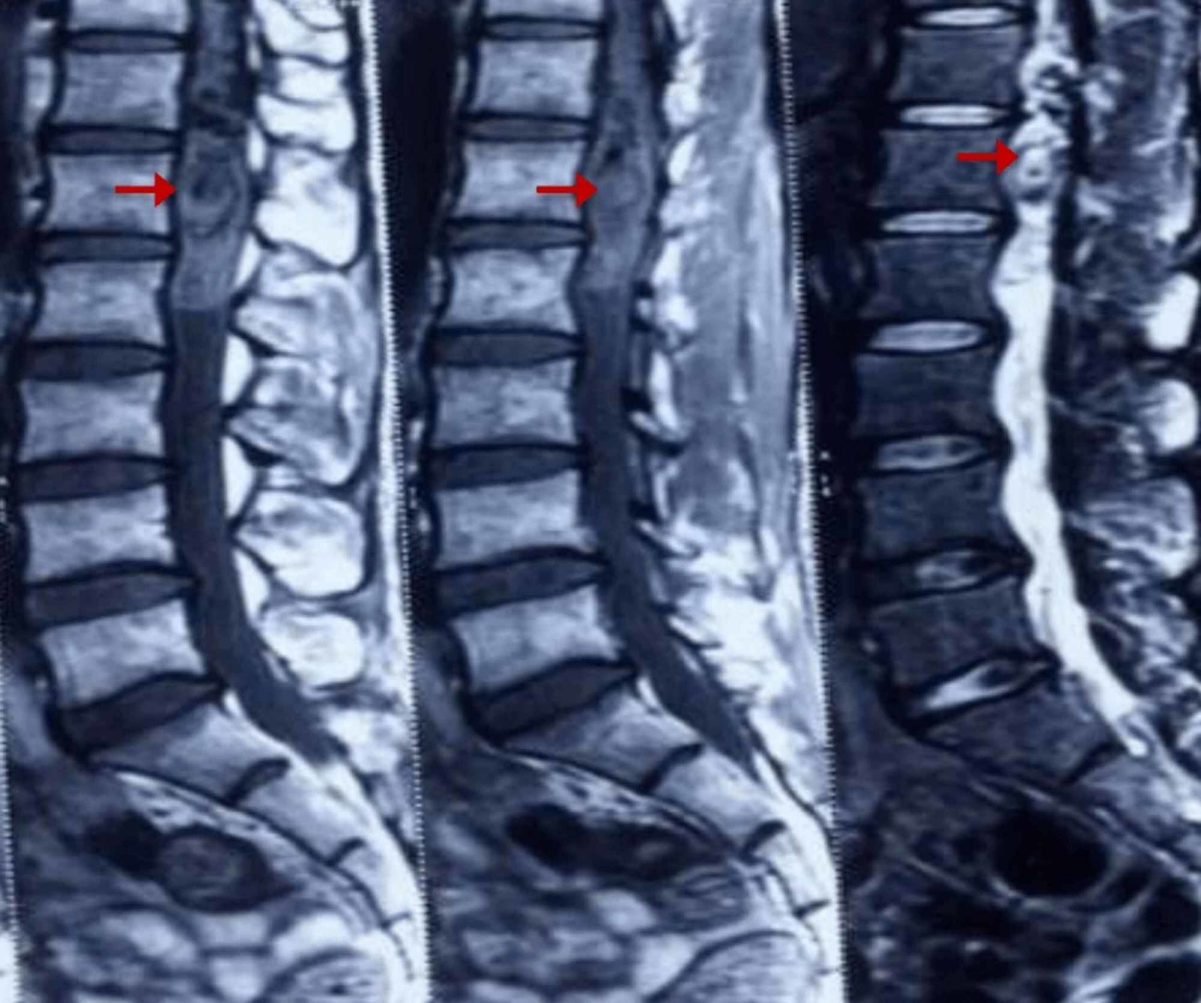
MRI lumbar spine of the patient The image shows intramedullary hemorrhage in the cord with mural nodule opposite D12 L1, which is intensively enhancing on contract suggestive of dural and pial arteriovenous malformations with intramedullary hemorrhage (arrows) MRI: magnetic resonance imaging

At the age of 29, the patient again presented with back pain radiating to the right lower limb, which was gradually progressive. On investigation, the MRI lumbar spine was suggestive of multiple flow voids along the posterolateral surface of the cord (Figure 1).

The patient was diagnosed with hemangioblastoma with dural arteriovenous malformations (AVM), for which he was operated on in 2011, and his back pain was relieved. A summary of the second incidence is shown in Table 1.

**Table 1:** Summary of the second incidence: patient diagnosed with hemangioblastoma AVM: arteriovenous malformation

Summary	Hemangioblastoma with dural arteriovenous malformations
Symptoms	Severe back pain radiating to right lower limb
Investigation	The scan indicated intramedullary hemorrhage in the cord with mural nodule opposite T12-L1, which was intensively enhancing on contract. It was suggestive of dural and pial AVM with intramedullary hemorrhage
Treatment	Operated for hemangioblastoma

Third Incidence

**Figure 2:**
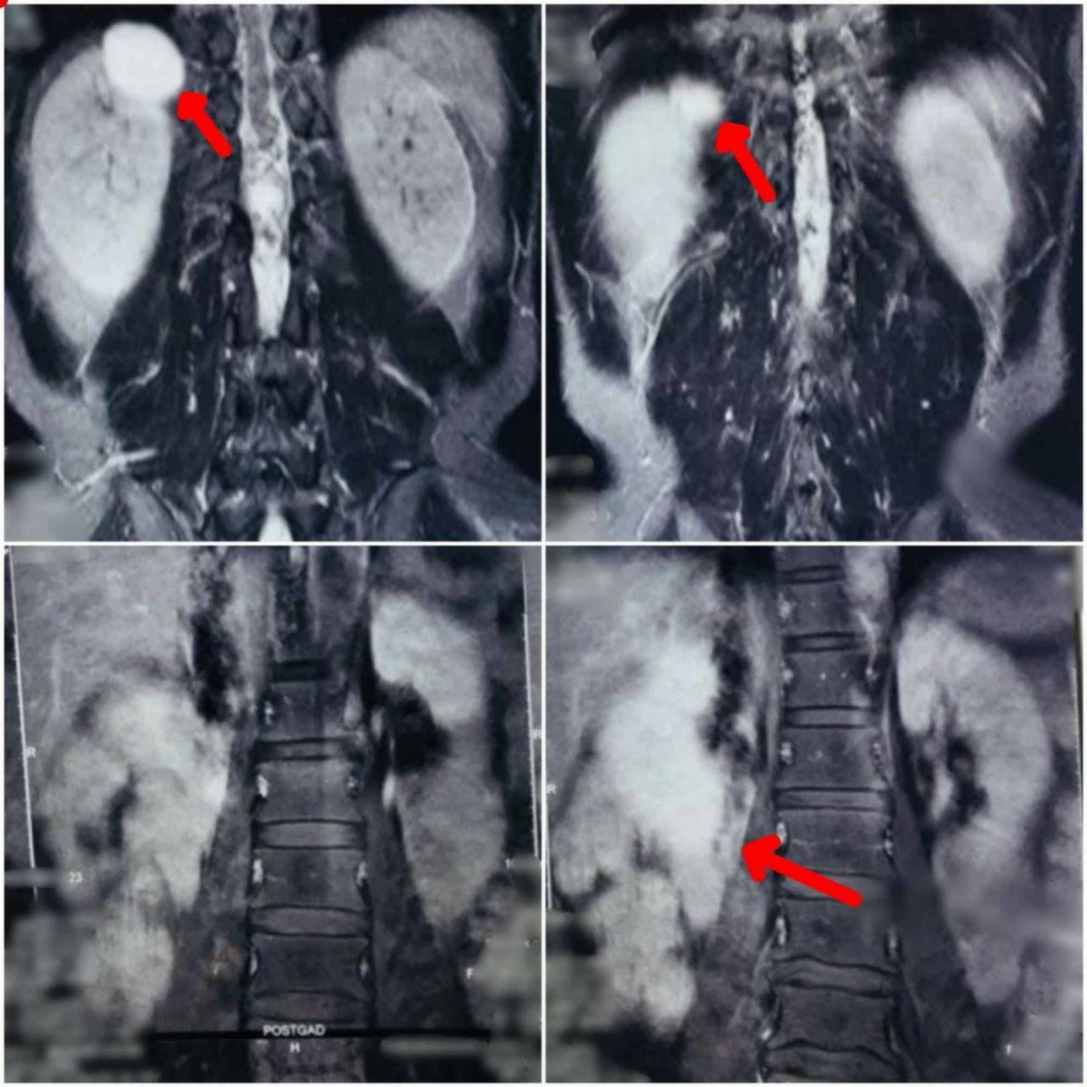
CECT abdomen during the third incidence The image shows bilateral heterogeneously enhancing suprarenal mass suggestive of bilateral pheochromocytoma (arrows on top). The scan also shows a heterogeneously enhancing right lobe of liver suggestive of either fibrolamellar hepatocellular carcinoma or metastatic tumor (arrow at bottom right) CECT: contrast-enhanced computed tomography

In 2015, during a postoperative follow-up with a neurosurgeon, the patient complained of severe pain in the abdomen accompanied by hypertension. Contrast-enhanced CT (CECT) abdomen was suggestive of bilateral heterogeneously enhancing suprarenal mass likely to be bilateral pheochromocytoma, and a heterogeneously enhancing right lobe of liver suggestive of either fibrolamellar hepatocellular carcinomas or metastatic tumor (Figure 2).

On further investigation, the metaiodobenzylguanidine (MIBG) scan revealed I-131 MIBG concentrating primary tumor in the bilateral suprarenal region. Hence, the patient was diagnosed with bilateral pheochromocytoma with liver metastasis. The patient underwent bilateral adrenalectomy with resection of segment six of the liver. Histopathology of the specimen of left adrenal mass and right adrenal mass gave an impression of bilateral pheochromocytoma with liver metastasis (chromogranin-positive, MIB1 <1%). A summary of the third incidence is shown in Table 2.

**Table 2:** Summary of the third incidence: patient diagnosed with bilateral pheochromocytoma with liver metastasis CECT: contrast-enhanced computed tomography; MIBG: metaiodobenzylguanidine

Summary		Bilateral pheochromocytoma with liver metastasis
Symptoms		Severe abdominal pain with hypertension
Diagnosis	1) CECT scan of the abdomen	Suggestive of heterogeneously enhancing suprarenal mass likely bilateral pheochromocytoma and a heterogeneously enhancing right lobe of liver suggestive of either fibrolamellar hepatocellular carcinoma or metastatic tumor
	2) MIBG scan	It revealed I-metaiodobenzylguanidine concentrating primary tumor in the bilateral suprarenal region; hence the patient was diagnosed with bilateral pheochromocytoma with liver metastasis
Treatment		Bilateral adrenalectomy with resection of segment six of the liver was done. Findings were: 1) 5 x 5-cm left suprarenal lump; 2) 4 x 4-cm right suprarenal mass; 3) firm growth in segment six of the liver; 4) mass of size 3 x 3 cm on right upper pole renal cyst

Fourth Incidence

**Figure 3:**
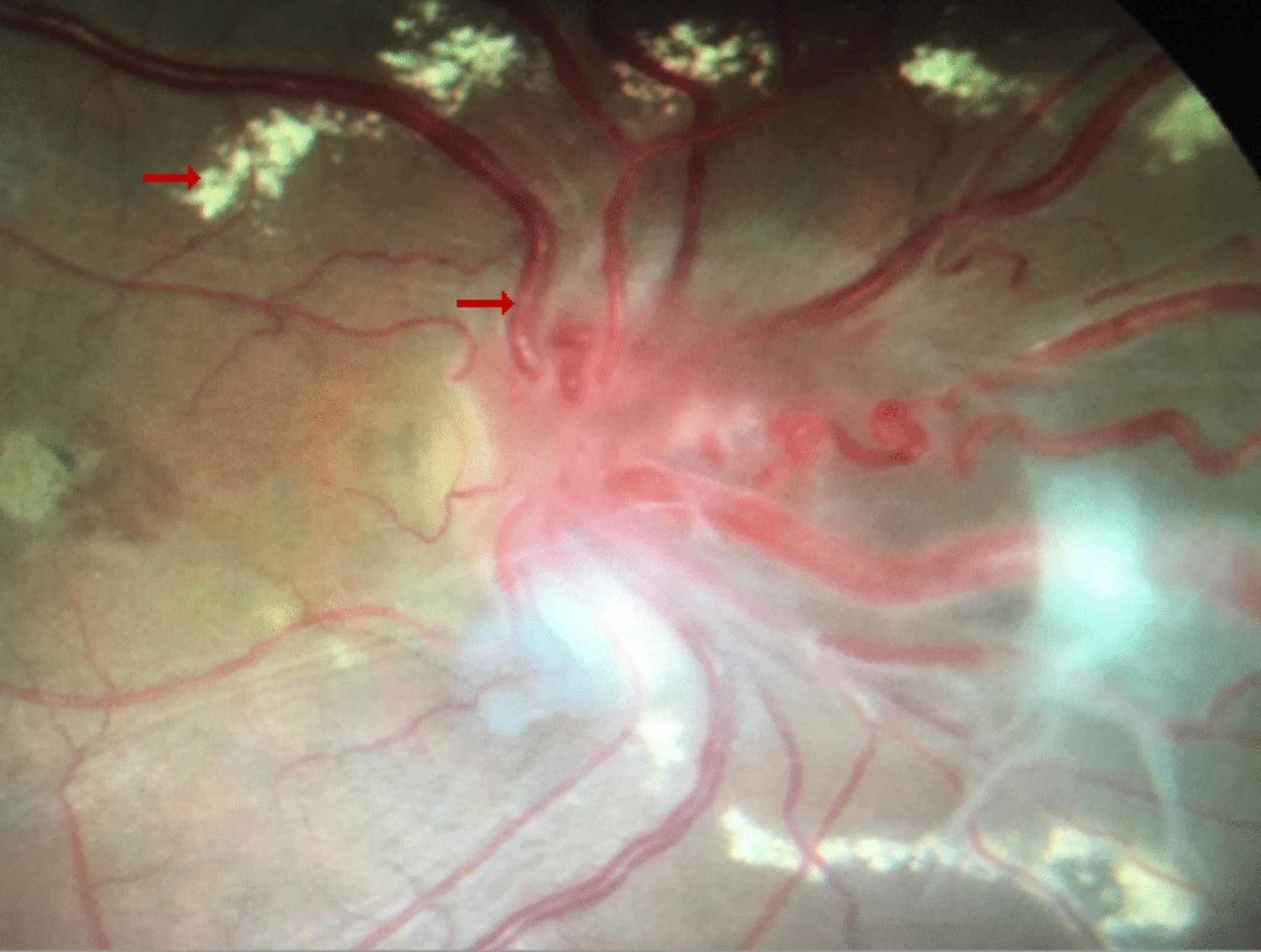
Fundus angiogram during the fourth incidence Angiogram shows right disc hyperemic with macular degeneration (top arrow), exudative retinal detachment nasal to disc, multiple exudates and secular dilation nasal to disc suggestive of retinal angiomas (bottom arrow)

In 2017, at the age of 36, the patient presented with a complaint of severe headache and cervical pain with light-headedness on walking. The fundus angiogram showed right disc hyperemic with macular degeneration and exudative retinal detachment nasal to disc. Multiple exudates and secular dilatation were present nasal to disc (Figure 3).

Triple-phase CT, and MRI of the brain, cervical, and lumbar spine along with mutation testing for VHL were done given the patient's history. Brain MRI showed a small nodular enhancing lesion of 3 x 1 mm adjacent to the left cerebellar peduncle; in the left cerebellar hemisphere, an enhancing lesion of 4 x 2 mm was seen. While visualizing the spinal cord, in the cervical region, heterogeneously enhancing lesion of 9.5 x 4.3 mm was noticed in an extramedullary location at the cervical vertebra-2 level along the right anterolateral aspect of the spinal canal and mildly extending into the right cervical vertebra-2 and 3 neural foramina. A small punctate enhancing lesion was seen along the right postero-lateral aspect, closely applied to the pial surface of the cord at the cervical vertebra 3-4 level. Evidence of past laminectomy was seen on the MRI. The scan revealed a heterogeneous lesion of 39 x 15 mm with solid, cystic/necrotic components, and heterogeneous intralesional and peripheral enhancement on post-contrast scans at lumbar vertebra 1-2 levels closely applied to the pial surface of lower dorsal cord in the conus and displacing them posteriorly. Multiple vascular flow voids were seen adjacent to lesions extending up to the lumbar region on caudal aspects and lower dorsal region on cranial aspects. Another small punctate enhancing lesion was seen along the nerve root of cauda equina at the lumbar vertebra 4-5 level. Subtle edema was also seen in the conus.

CECT abdomen was suggestive of postoperative B/L pheochromocytoma, adrenalectomy, and liver lobectomy. A heterogeneously enhancing lesion with the cystic component in the spinal canal component was observed, suggesting hemangioblastoma. Sequencing of the VHL gene revealed a heterozygous VHL gene with a peak height of mutant allele smaller, which was suggestive of mosaicism for the mutant allele. A summary of the fourth Incidence is given in Table 3.

**Table 3:** Summary of the fourth Incidence: patient diagnosed with multiple tumors CECT: contrast-enhanced computed tomography; MRI: magnetic resonance imaging; VHL: Von Hippel-Lindau

Summary		Multiple hemangioblastomas at various levels in the brain and spinal cord
Symptoms		Severe headache and cervical pain with light-headedness on walking
Diagnosis	1) Retinal angiogram	Right disc hyperemic with macular degeneration and exudative retinal detachment nasal to disc was seen
	2) MRI of the brain and spinal cord	Multiple heterogeneously and punctate enhancing lesions were seen on post-contrast scans
	3) CECT of the abdomen	Showing postoperative bilateral pheochromocytoma adrenalectomy and liver lobectomy. Also showing the right renal cortical cyst
	4) VHL gene sequencing	Result of sequencing was heterozygous VHL gene

Based on the above findings and the patient’s history of all the four incidences, the patient was diagnosed with VHL syndrome.

Case 2

First Incidence

A 25-year-old Southeast Asian male presented with a history of vision loss in 2007. At that time, he was given the diagnosis of retinal hemangioblastoma (RHB). Later, he had multiple retinal surgeries, which improved his vision. At the same time, his sequencing of the VHL gene came back positive. However, he did not follow up on it after the surgery.

Second Incidence

**Figure 4:**
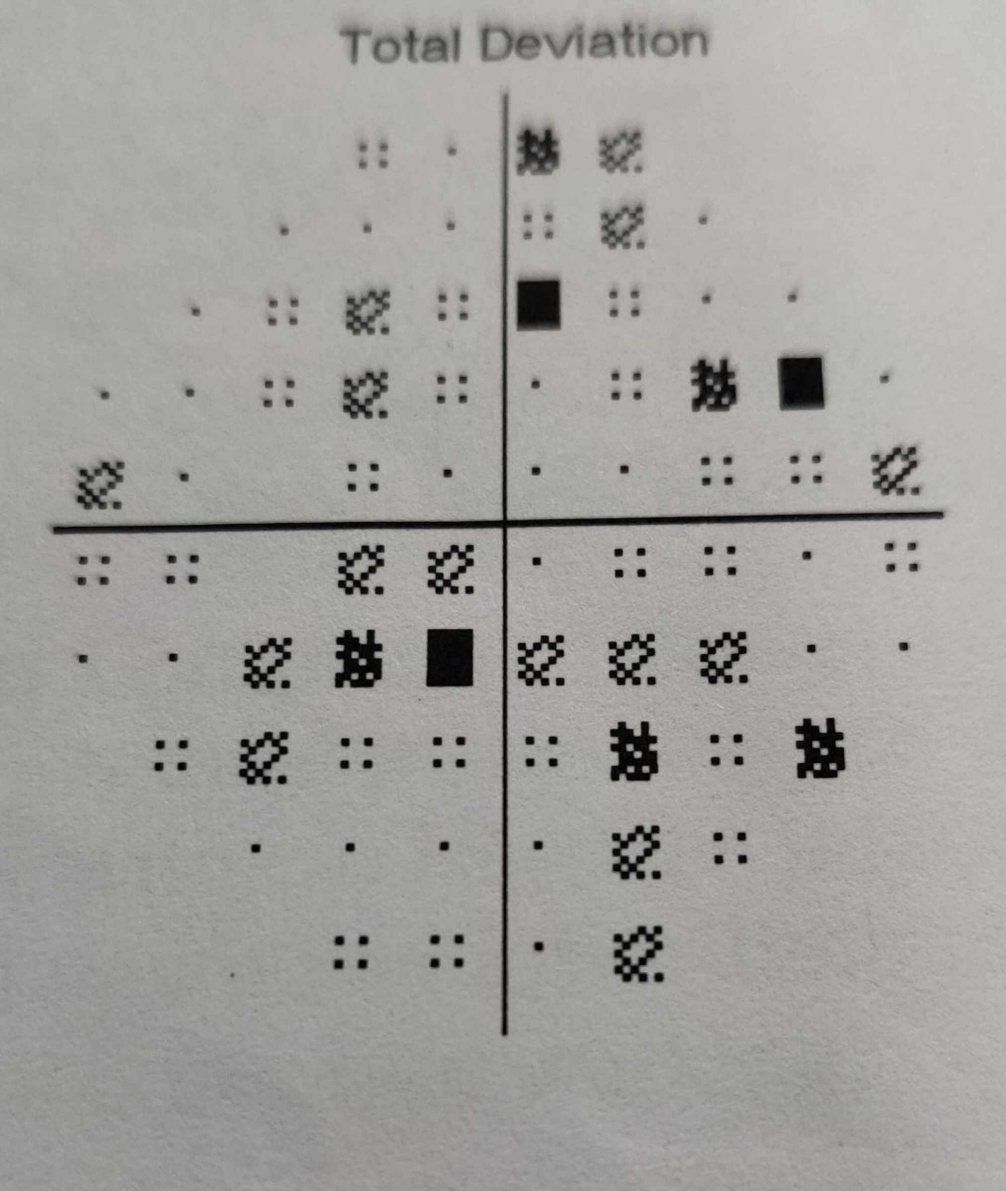
Perimetry image shows enlargement of blind spot with few scotomas

In 2013, he suffered left eye vision loss. A perimetry test showed scotomas on perimetry with an enlarged blind spot (Figure 4). 

**Figure 5:**
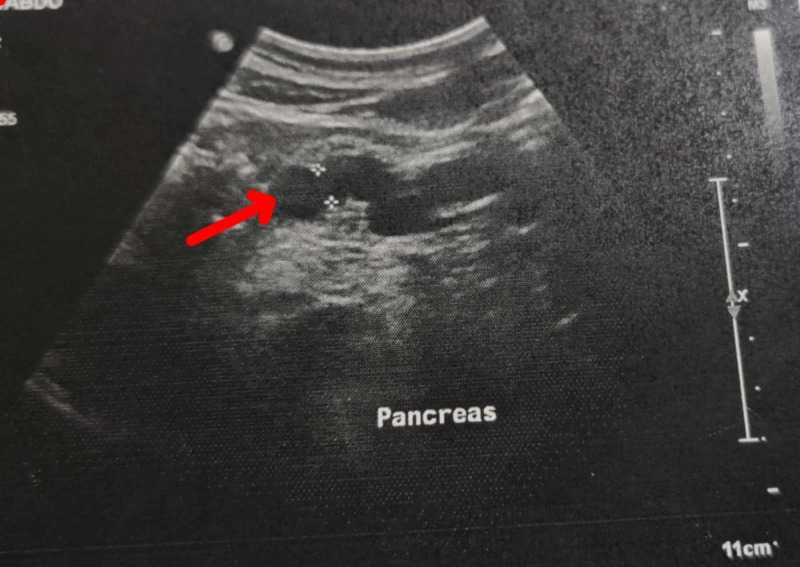
Ultrasonography of the patient The image shows evidence of chronic pancreatitis (arrow)

Within a year after this presentation, he complained of headaches with intermittent dizziness and incoordination. On clinical examination, he was found to have right upper and lower limb numbness with gait ataxia. On further investigation, the MRI brain was suggestive of a cystic lesion with midline posterior fossa tumor, compressing anterior and posterior surface medulla oblongata with resultant hydrocephalus. Later on, he was operated for posterior fossa tumor with cervical-1 arch tumor excision, which was sent for excision biopsy and came back with results suggestive of hemangioblastoma. He then developed pain in his abdomen during the same admission; his ultrasound abdomen was done, which showed chronic pancreatitis with distal calculus (Figure 5). He responded to symptomatic therapy with hydration. After that, he remained healthy for six years until the present admission.

Third Incidence

**Figure 6:**
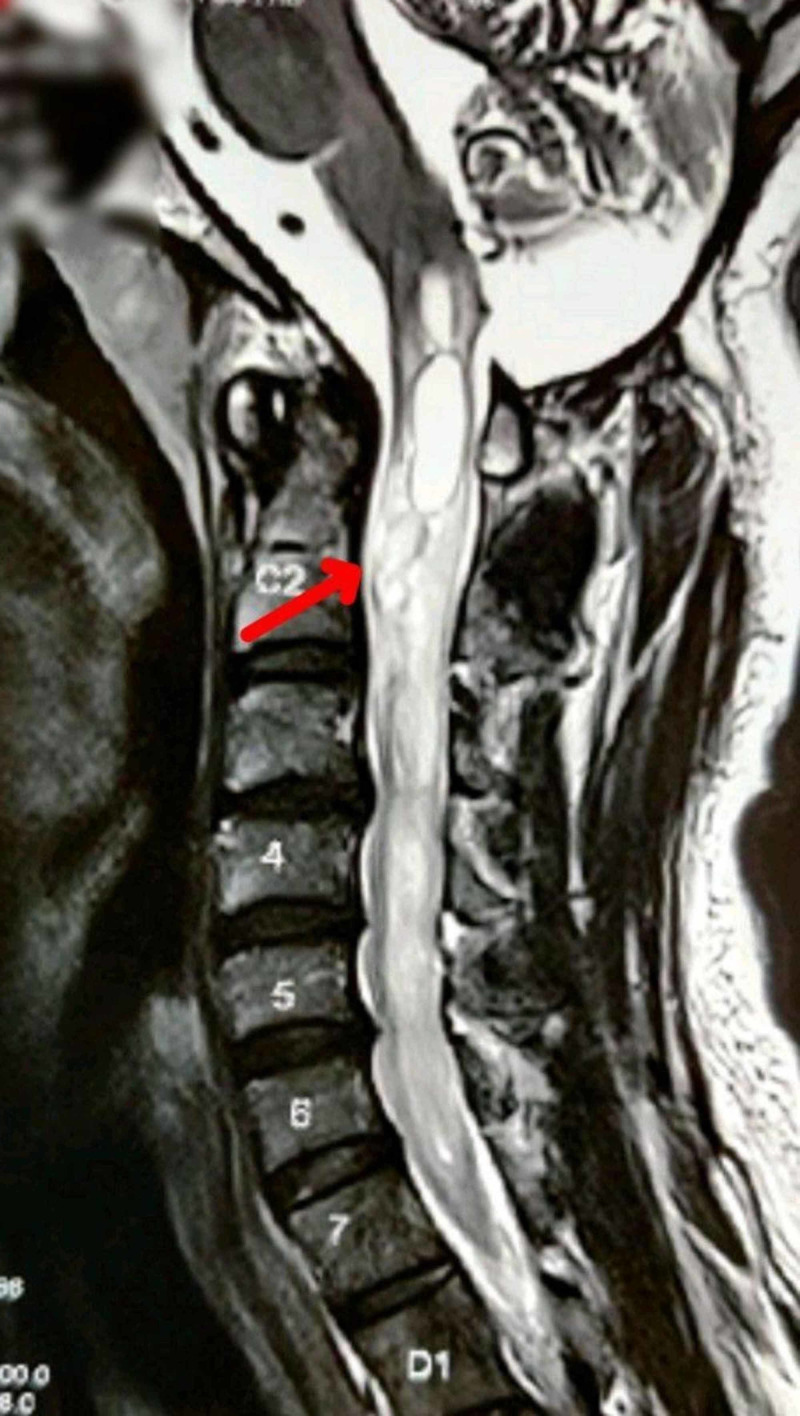
MRI – T2-weighted image showing sagittal section of cervicomedullary junction The image shows long heterogeneous cystic lesion starting from lower medulla through the whole cervical cord, up to the seventh cervical level, suggestive of hemangioblastoma (arrow) MRI: magnetic resonance imaging

**Figure 7:**
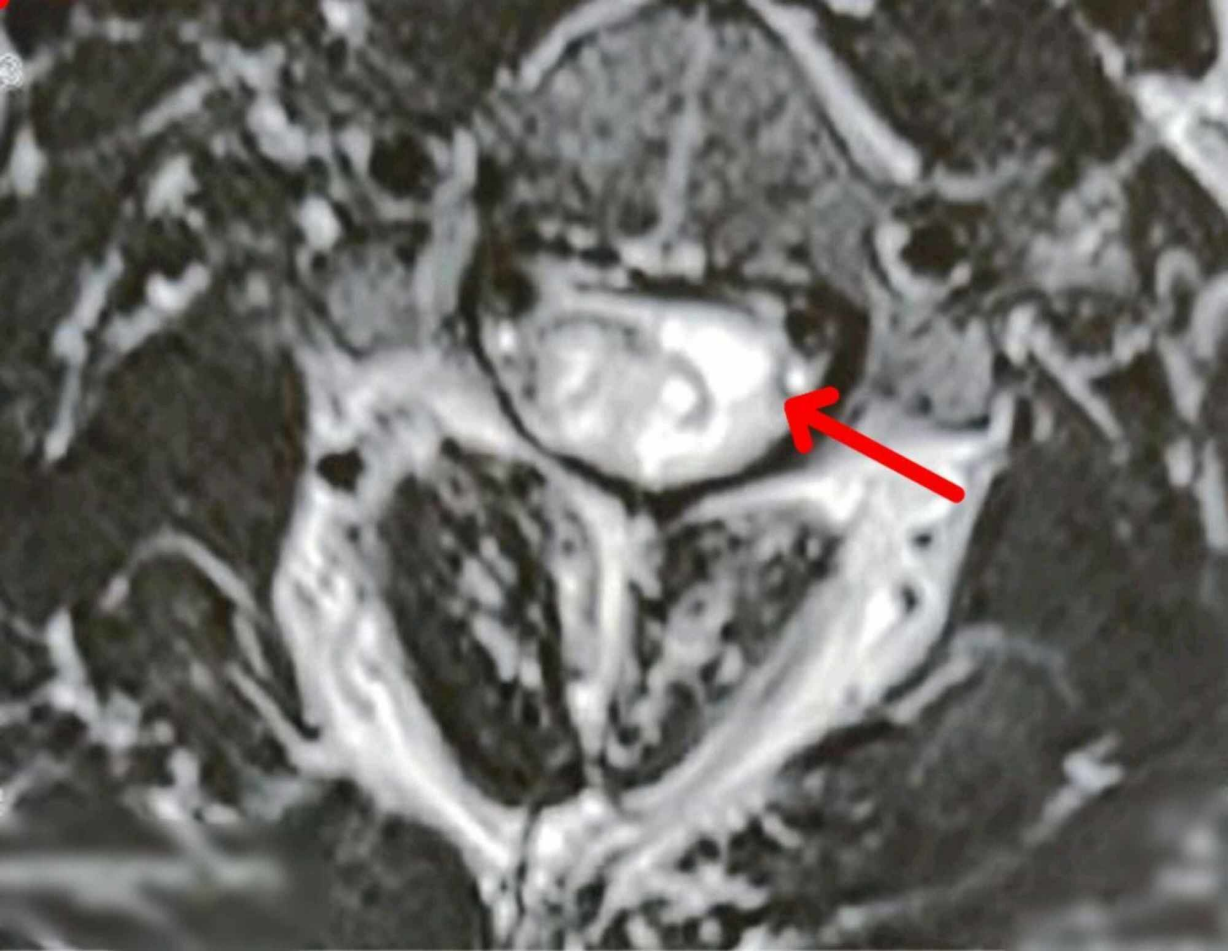
MRI – weighted axial image of the cervical spine The image shows a cystic lesion on the left side of the cervical cord, suggestive of hemangioblastoma (arrow) MRI: magnetic resonance imaging

In March 2020, at the age of 38, the patient presented with complaints of neck pain and difficulty walking for the past one month. Considering his symptoms and past medical history, we performed a cervical spine MRI, which showed a heterogeneous cystic lesion in the cervical cord starting from lower medulla till upper dorsal spine, suggestive of hemangioblastoma (Figures 6, 7). A formal diagnosis of VHL syndrome was made. A summary of the case is shown in Table 4.

**Table 4:** Summary of manifestations in the second case in the span of a few years

Year of incidence	Distinct manifestations occurring over the years (2007-2020)
2007	Occurrence of retinal hemangioblastoma
2014 (November)	Cystic lesion with midline posterior fossa tumor compressing anterior and posterior surface medulla oblongata suggestive of hemangioblastoma
2014 (November)	Chronic pancreatitis with distal calculus
2020 (March)	Heterogeneous cystic lesion suggestive of hemangioblastoma in cervical cord starting from lower medulla till upper dorsal spine

## Discussion

VHL syndrome is a rare disorder with a prevalence rate of around three out of 100,000 births per year. It is associated with an extensive range of symptoms and is difficult to diagnose early [6]. VHL is diagnosed when a patient has the following combination of genetic or family factors: 1) at least two CNS hemangioblastomas; 2) at least one CNS hemangioblastoma and one other manifestation described above; 3) at least one of the manifestations described above and a pathogenic mutation in the VHL gene or a first-degree relative with VHL [9].

The most prevalent tumor in VHL syndrome is hemangioblastoma. CNS hemangioblastoma can be observed in nearly 72% of patients with VHL. These patients usually present with headaches, problems with balance and walking, dizziness, and weakness of the limbs. Both of our patients had spinal and brain hemangioblastoma at some stages in their lives. However, there is no modality for precise and adequate management of hemangioblastomas; patients suffering from inoperable hemangioblastomas or with high surgical complication rates are generally treated with either standard split radiation therapy or stereotactic radiation therapy, which have demonstrated positive results [10]. Tampieri et al. have indicated that if hemangioblastomas are small, they can be cautiously managed with radiological follow-up, and operations should only be carried out in patients with symptomatic hemangioblastomas [11]. Surgical outcomes are usually excellent, with no documented recurrence after surgery, but other regions of the CNS are at high risk of developing additional hemangioblastoma. Walther et al. have suggested that embolization is feasible in preoperative hemangioblastomas; this leads to decreased blood loss and fewer postoperative complications [12].

Pheochromocytoma is another critical feature in the clinical classification of VHL syndrome, and it has been seen that there is a strong association between pheochromocytoma and VHL syndrome [13]. It is usually found in young adults and can be adrenal or extra-adrenal. There may be a lack of typical symptoms of pheochromocytoma, which are tachycardia, diaphoresis, tachypnea, severe headache, angina, cold and clammy skin, palpitations, nausea, vomiting or epigastric discomfort, and postural hypotension. These clinical signs were absent in about 35% of patients in a study, with 33 patients having VHL syndrome and 37 patients having pheochromocytoma [13].

The lack of such symptoms can make the detection of pheochromocytoma more challenging. Both biochemical and radiological evidence can be used for diagnosing pheochromocytoma. Blood samples are used to ascertain plasma epinephrine or norepinephrine; similarly, urine samples can detect vanillylmandelic acid [14]. The best way to treat pheochromocytoma in a VHL patient is by surgical resection; it has shown excellent results, and no recurrences have been reported [15]. Our first case presented with pheochromocytoma at the age of 34 years and later underwent bilateral adrenalectomy with resection of liver segments.

RHB, which is usually bilateral and multifocal or occurs over the years, is the first presenting event in around half of VHL patients [3,16]. Both of our patients presented with RHB at the age of 27 and 25 years respectively as a primary complication and both underwent retinal surgeries. Even though RHB is benign in nature and slow-growing, it can result in vision-threatening complications such as macular exudation, retinal traction, retinal detachment, vitreous hemorrhage, neovascular glaucoma, and phthisis [17]. These are more commonly seen in the peripheral retina. Coat's disease, retinal macroaneurysm, cavernous retinal hemangioma, racemose hemangioma, and vasoproliferative tumors are included in the differential diagnosis of RHB. Fluorescein angiography has a crucial role in tracking, localizing, and evaluating small RHBs. In patients with VHL syndrome, visual prognosis is often poor, but early recognition and diagnosis can modify visual prognosis [18]. The various manifestations related to VHL's multisystem involvement are shown in Table 5 [9].

**Table 5:** Manifestations included in the criteria for the clinical diagnosis of Von Hippel-Lindau syndrome

Serial number	Manifestations
1.	Central nervous system hemangioblastoma
2.	Retinal hemangioblastoma
3.	Renal cell carcinoma
4.	Pheochromocytoma, paraganglioma, and/or glomus tumor
5.	Neuroendocrine neoplasm and/or multiple cysts of the pancreas
6.	Endolymphatic sac tumor

**Figure 8:**
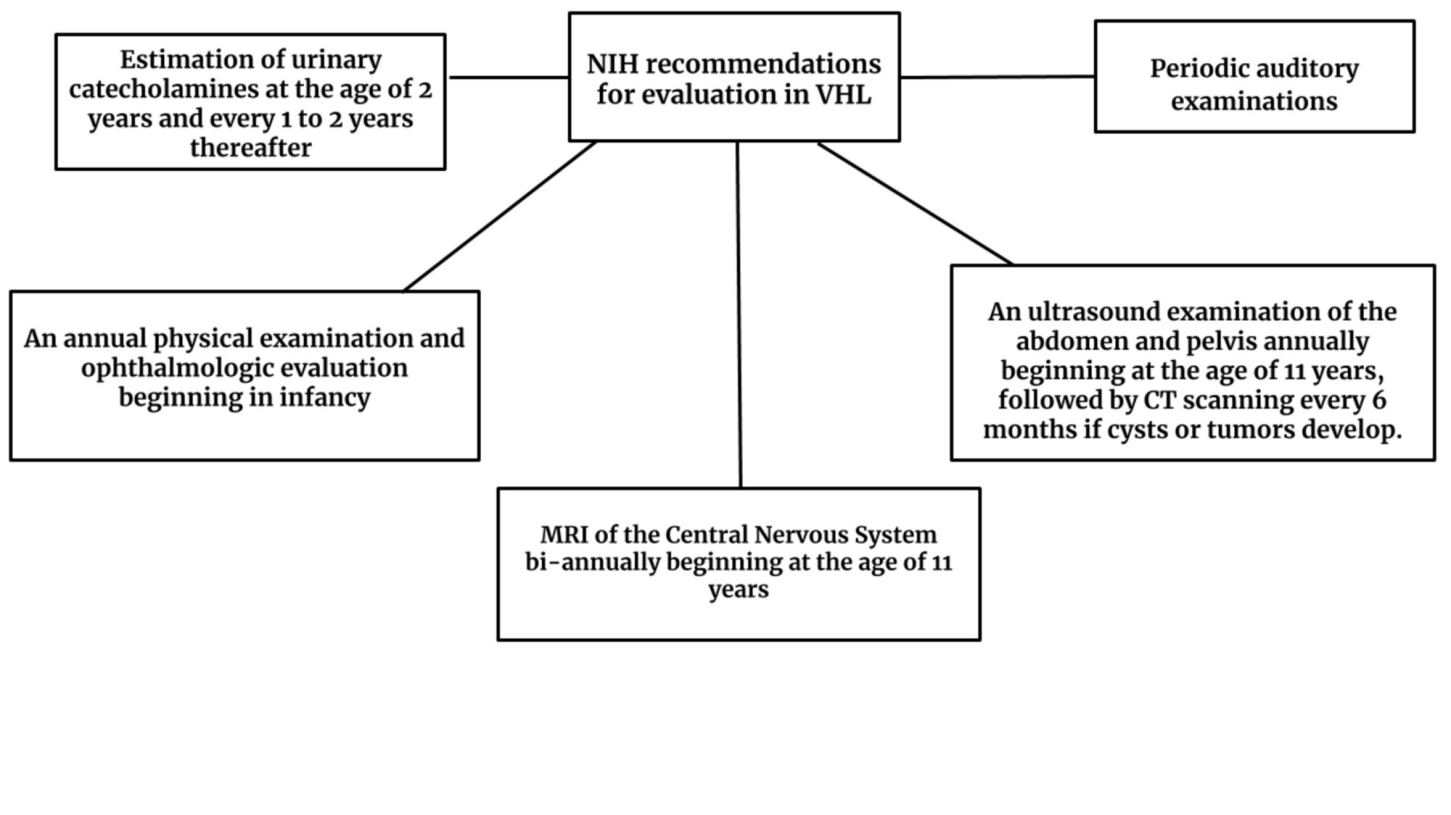
National Institute of Health (NIH) recommendations for evaluation in Von Hippel-Lindau syndrome VHL: Von Hippel-Lindau disease; CT: computed tomography; MRI: magnetic resonance imaging

VHL should be taken into account in all patients with early-onset or multifocal RCC or RCC associated with one or more of the following conditions: family history of blindness, a history of visual or neurological symptoms; or renal cancer; CNS tumors, or coexistent pancreatic cysts, or inner-ear tumors, and epididymal lesions [7,19]. Relatives of patients with documented VHL should consider genetic evaluation. National Institute of Health (NIH) recommendations for the periodic evaluation in VHL are shown in Figure 8 [20].

## Conclusions

VHL syndrome is a rare genetic disorder that is associated with considerable rates of morbidity and mortality in adults. Early diagnosis can aid in understanding and treating this disorder more effectively. However, reaching a prompt diagnosis of VHL is a challenge, as it is a rare syndrome that involves several organ systems, including CNS, endocrine and renal systems, and eyes. A timely and detailed management plan would allow the prevention of complications and negative outcomes. Surgical excision may be used as a treatment option for various tumors if diagnosed in their preliminary stages. Periodical clinical follow-up for a sustained period of time is important in the management of these patients.
